# Epidemiological characteristics and temporal-spatial clustering analysis on human brucellosis in Jiangsu Province, 2006–2021

**DOI:** 10.1038/s41598-023-46690-z

**Published:** 2023-11-16

**Authors:** Nan Zhang, Xin-yu Fang, Wei-zhong Zhou, Zhong-ming Tan, Shu-yi Liang, Xiao-chen Wang, Jian-li Hu, Chang-jun Bao, Wen-dong Liu

**Affiliations:** 1 Department of Acute Infectious Diseases Control and Prevention, Jiangsu Provincial Centre for Disease Control and Prevention, 172, Jiangsu Rd, Nanjing, 210009 Jiangsu Province China; 2 Department of Food Safety and Evaluation, Jiangsu Provincial Centre for Disease Control and Prevention, 172, Jiangsu Rd, Nanjing, 210009 Jiangsu Province China

**Keywords:** Infectious diseases, Space physics

## Abstract

The marked increase in the incidence rate of brucellosis is a serious public health concern in Jiangsu Province. However, its temporal and spatial distribution has not been studied in depth. The main purpose of this study is to depict the demographic, temporal and spatial distribution patterns and clustering characteristics of brucellosis cases in Jiangsu Province, China, from 2006 to 2021 to develop and implement effective scientific prevention and control strategies. Data for human brucellosis cases in Jiangsu Province from 2006 to 2021 were obtained from the Nationwide Notifiable Infectious Diseases Reporting Information System (NIDRIS). Spatial autocorrelation analysis and temporal-spatial scan statistics were used to identify potential changes in the spatial and temporal distributions of human brucellosis in Jiangsu Province. During the years 2006–2021, 1347 brucellosis cases were reported in Jiangsu Province, with an average annual incidence rate of 0.1036 per 100,000 individuals. Middle-aged and elderly individuals (aged 40–69 years) were the main infected populations, accounting for 69.72% (939/1347) of all reported cases. The incidence of brucellosis in Jiangsu showed a long-term increasing trend and displayed pronounced seasonal variations, with the peak occurring between April and June annually. The incidence gradually expanded from the northern and southern areas to the central areas between 2006 and 2021. Global spatial autocorrelation analysis demonstrated a positive correlation in the incidence of brucellosis between 2008 and 2012–2021. Temporal-spatial clustering analysis showed that the primary cluster was detected in the northern, highly endemic regions of Jiangsu, and the three secondary clusters were in areas where there had been outbreaks of brucellosis. Human brucellosis remains a serious public health issue in Jiangsu Province. Northern and southern Jiangsu regions, with high rates of brucellosis, may require special plans and measures to monitor and control the disease. Additionally, the capacity to respond to outbreaks in high-incidence areas should be improved to prevent further brucellosis outbreaks.

## Introduction

Brucellosis is a zoonotic disease caused by infectious bacteria of the genus *Brucella*, which accounts for a minimum of 500,000 human brucellosis cases worldwide each year. This disease not only causes severe physical and mental damage and even disability, but is also difficult to treat in chronic patients, increasing social healthcare costs and depleting medical resources. Low-and middle-income countries are considered high-prevalence regions^1^. Zoonotic brucellosis is often caused by various *Brucella* species, 12 of which are now recognised. Among these, *B. melitensis* is the most prominent zoonotic agent, followed by *B. abortus* and *B. suis*. The occurrence of brucellosis in humans is directly related to the prevalence of the disease in animals living in a particular geographic region^2,3^. The main sources of *Brucella* infections in humans are sheep, goats, cattle/other *Bovidae,* and pigs. Human infection with brucellosis primarily occurs through direct or indirect contact with infected domestic animals and contaminated materials via the skin, digestive tract, respiratory system, and mucosa^4,5^. The brucellosis epidemic not only poses a significant threat to human health but also hinders livestock production, thereby affecting the global economy and trade^6,7^. Additionally, it causes problems in food safety^8^, which is an important public health concern worldwide, particularly in developing countries^9^. According to data released by the Chinese Bureau for Disease Control and Prevention, the incidence of brucellosis in China has been on an overall upward trend between 2006 and 2021, and while the incidence decreased between 2016 and 2018, it increased every year from 2019 to 2021. In 2021, the number of reported cases (69,767) and incidence rate (4.95/100,000) reached their highest recorded values, with brucellosis cases reported in 31 provinces (autonomous regions) in China^10^, making it a significant public health concern in the country. The intensity of the brucellosis epidemic in Jiangsu Province is consistent with that of China.

In recent years, human brucellosis has become highly endemic in China and shows distinct temporal-spatial aggregation. Brucellosis has been mainly concentrated in northwestern and northeastern China over time but has further expanded to central and southern regions^11^. Previous studies on brucellosis in Jiangsu Province have focused on epidemiological descriptions and analyses; however, its temporal and spatial distributions have not been studied in depth. This study aims to provide a scientific basis for adjusting prevention and control strategies and recommendations by systematically analysing the epidemic characteristics of human brucellosis and exploring the temporal-spatial aggregation characteristics in Jiangsu Province from 2006 to 2021.

## Materials and methods

### Criteria for laboratory diagnosis

Brucellosis was diagnosed according to the Diagnostic Criteria for Brucellosis (WS269-2019). Laboratory-diagnosed cases of brucellosis are defined as follows: Follow-up laboratory testing should be performed for individuals with a history of *Brucella* exposure or clinical symptoms of brucellosis, or both. If a person tests positive for the Rose Bengal Plate Agglutination Test (RBT) and the Tube Agglutination Test (SAT: serum agglutination test: a titre of 1:100 +  + or above, or a titre of 1:50 +  + or above for patients with a disease lasting more than one year and still showing clinical symptoms), or if *Brucella* is isolated from the culture of any of the pathological materials such as the patient's blood, bone marrow, other body fluids, or excreta, then the individual can be confirmed as a case of brucellosis.

### Data source

**Figure 1:**
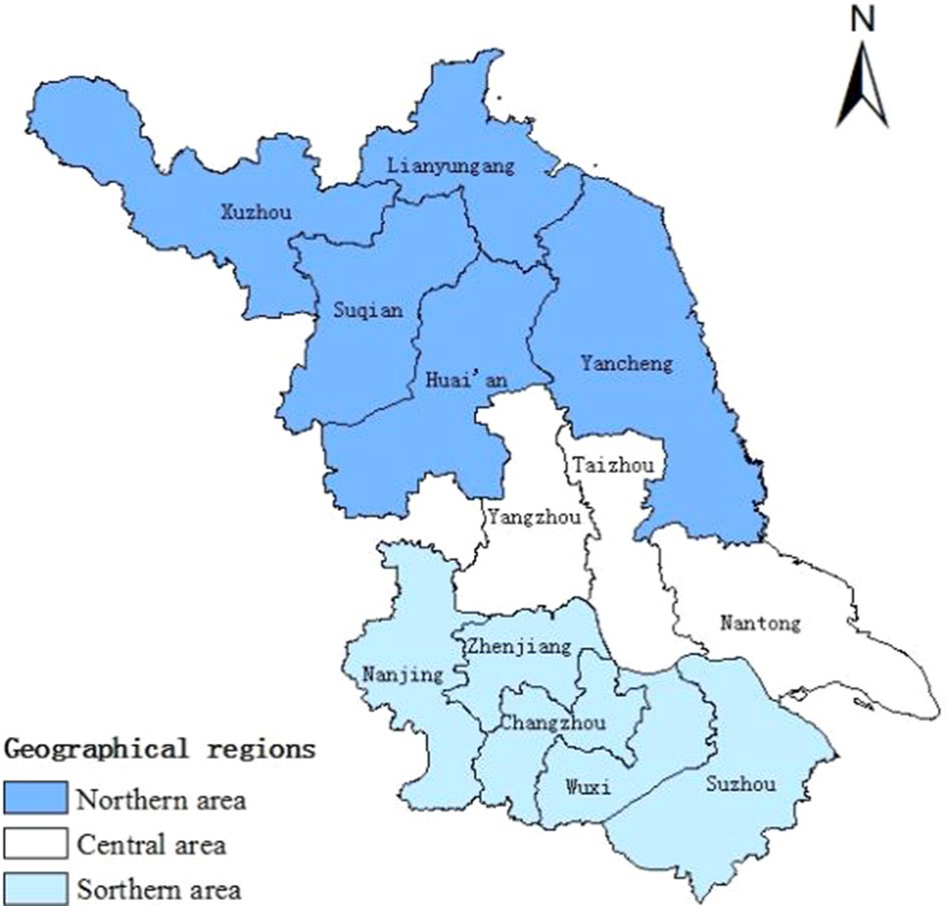
Three geographical regions of Jiangsu Province.

Human brucellosis is a notifiable Class B infectious disease in China. Once diagnosed, cases must be reported to the Nationwide Notifiable Infectious Diseases Reporting Information System (NIDRIS)^12^. The NIDRIS was established in 2004 and covers all healthcare institutions across China. In this study, we obtained human brucellosis data from 2006 to 2021 in Jiangsu Province from the NIDRIS. Demographic data for the corresponding periods were obtained from the Jiangsu Provincial Bureau of Statistics. The digital vector map of Jiangsu Province was obtained from the National Geographic Information Resources Directory Service System (https://www.webmap.cn). Jiangsu Province has 13 cities with jurisdiction over 107 counties (urban areas). To analyse the spatial distribution characteristics of brucellosis in Jiangsu province, in this study, the cities were divided into three geographical regions: southern area (Nanjing, Suzhou, Wuxi, Changzhou and Zhenjiang), central area (Yangzhou, Taizhou and Nantong), and northern area (Xuzhou, Lianyungang, Suqian, Huai'an and Yancheng), these three regions are shown in Fig. 1. In this study, “County” in Jiangsu Province was selected as the research unit.

### Global spatial autocorrelation analysis

Spatial autocorrelation analysis methods include global and local spatial autocorrelations. The Moran index (Moran's *I*) for global spatial autocorrelation was proposed by Moran in 1948^13^. This reflects the degree of similarity of the unit attribute values in adjacent regions of space. Equation (1) was used to calculate Moran's *I,* based on the covariance of the statistical correlation coefficients^14^.

1 $$I = \frac{n}{{\mathop \sum \nolimits_{i = 1}^{n} \mathop \sum \nolimits_{j = 1}^{n} W_{ij} }} \times \frac{{\mathop \sum \nolimits_{i = 1}^{n} \mathop \sum \nolimits_{j = 1}^{n} W_{ij} \left( {X_{i} - \overline{X}} \right)\left( {X_{j} - \overline{X}} \right)}}{{\mathop \sum \nolimits_{i = 1}^{n} (X_{i} - \overline{X})^{2} }}$$

*W*_*ij*_ is the spatial weight matrix reflecting the spatial scale changes of adjacent regions and ${X}_{i}, \overline{X }$. Moran’s *I* > 0 indicates a positive correlation, Moran’s *I* < 0 indicates a negative correlation, and Moran’s *I* = 0 indicates that the regionalised variables are randomly distributed in space. The equation for the standardisation of the *I* value is: $Z(I) = [I - E(I)]/\sqrt {Var(I)}$, and is used to test for the existence of spatial autocorrelation. When |*Z*| > 1.96, *p* < 0.05, it was considered statistically significant, indicating spatial autocorrelation^14^. In this study, global spatial autocorrelation was used to describe the overall spatial distribution pattern of brucellosis in the Jiangsu Province annually from 2006 to 2021.

### Temporal-spatial scan statistics

Retrospective temporal-spatial scan statistics proposed by Kulldorff^14^ were used to determine whether brucellosis cases were randomly distributed over space and time in the study area from January 2006 to December 2021. This method uses a cylindrical window with a circular geographic base and height corresponding to the time dimension. In this way, it is possible to move in space and time with variable sizes and locations, scanning not only the entire study area but also covering the entire study period, in principle, using an infinite number of scanning windows. The null hypothesis assumes that the relative risk (RR) of brucellosis is the same within the window compared with outside it, while the alternative is that the RR is elevated within the window compared to the outside. Assuming that brucellosis cases follow a discrete Poisson distribution on spatial and temporal scales^15^, the likelihood ratio (LLR) for a specific window is calculated as follows^16^:

2 $$LLR = {\text{log}}\left( {{\text{c}}/{\text{n}}} \right)^{{\text{c}}} \left[ {\left( {{\text{C}}{-}{\text{c}}} \right)/\left( {{\text{C}}{-}{\text{c}}} \right)} \right]^{{({\text{C}}{-}{\text{c}})}}$$

where C represents the total number of cases, and c and n are the actual and theoretical numbers of cases in the scanning window, respectively. A *p*-value is estimated through Monte Carlo simulation to test the LLR (α = 0.05). The window with the maximum LLR was defined as the most likely cluster, that is, the cluster least likely to be due to chance. Other windows with statistically significant LLR are considered secondary clusters^17^.

In this study, the spatial size of the scanning window was limited to 40% of the total population at risk, the length of time was limited to 90 days, and the Monte Carlo simulation was repeated 999 times.

### Statistical analysis

Microsoft Excel 2010 software was used to establish a brucellosis database from 2006 to 2021 in Jiangsu Province. Descriptive statistics were employed to illustrate the epidemiological characteristics of human brucellosis in Jiangsu Province. Seasonal decomposition was applied to analyse incidence time series. ArcGIS 10.3 software was used for global spatial autocorrelation analysis and to map the spatial distribution and visual presentation of the clustering statistical results of human brucellosis. The SaTScan 9.4 software was used to conduct the spatiotemporal clustering analysis.

## Results

### Demographic characteristics

Human brucellosis was first reported in 2006 in Jiangsu Province. A total of 1347 cases occurred between 2006 and 2021, with an average annual incidence rate of 0.1036 per 100,000 individuals. The incidence rate in men was much higher than that in women, with a male-to-female ratio of 2.45:1. The age of the patients ranged from 3 months to 86 years, with a median age of 51 years. Middle-aged and older adults (40–69 groups) were the main infected populations, accounting for 69.72% (939/1347) of all the reported cases. Most affected individuals were farmers (59.61%, 803/1347), followed by individuals engaged in housework or facing unemployment (17.45%, 235/1347).

### Temporal distribution

**Figure 2:**
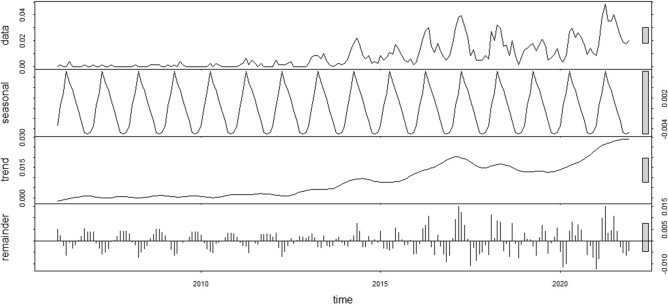
Seasonal and long-term trends of brucellosis in Jiangsu Province, 2006–2021.

As shown in Fig. 2, the incidence of brucellosis in Jiangsu showed a long-term increasing trend from 2006 to 2021. From 2006 to 2010, the incidence of brucellosis was low (the annual number of cases was less than 10, and the incidence range was 0.004/100,000–0.0078/100,000). The incidence has increased rapidly in recent years, with an average incidence rate of 0.2227 per 100,000 reported during 2016–2021 (ranging from 0.1727 per 100,000 in 2019 to 0.3363 per 100,000 in 2021), an increase of 265.72% compared with that of 0.0609 per 100,000 reported during 2011–2015(ranging from 0.0152 per 100,000 in 2012 to 0.1134 per 100,000 in 2014). The peak incidence occurred in 2021 (0.34/100,000). The results shown in Fig. 2 also demonstrate that brucellosis in Jiangsu Province exhibits significant seasonality. Most reported cases were observed between February and September, accounting for 82.93% (1117/1347) of the total cases, with the peak occurring annually between April and June. Conversely, the lowest number of cases occurred in October, and fewer cases were reported from October to January.

### Spatial distribution

The incidence of human brucellosis in Jiangsu Province gradually expanded from both the northern and southern areas to the central regions from 2006 to 2021. Brucellosis cases first emerged in Tongshan County of Xuzhou City in northern Jiangsu, and later in several counties within Xuzhou City (Suining, Xuzhou Gulou District, Yunlong District, Pizhou County, and Feng County), Sucheng of Suqian City, Guanyun of Lianyungang City in northern Jiangsu, Yixing of Wuxi City, Changzhou City (Liyang and Wujin), Huqiu of Suzhou City, and several other marginal counties in southern Jiangsu. Human brucellosis in Jiangsu has rapidly increased since 2011, and the affected regions have rapidly expanded from the northern and southern regions to Taizhou and Nantong in the central region. Brucellosis was an epidemic in all cities of Jiangsu until 2017, with the number of counties reporting cases increasing from 13 in 2006 to 58 in 2017.

**Figure 3:**
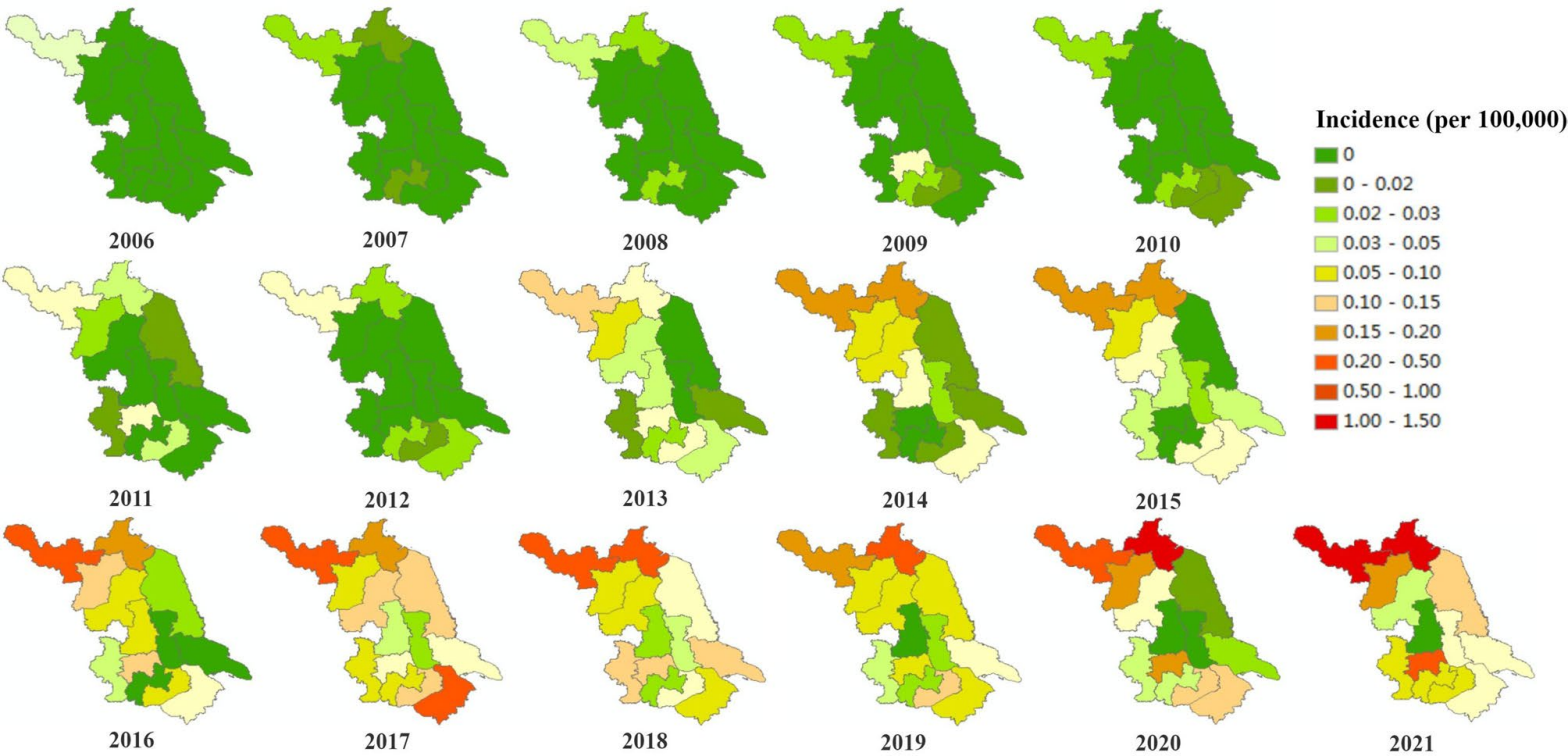
Temporal-spatial distribution map of brucellosis incidence in Jiangsu Province from 2006 to 2021.

The major brucellosis endemic regions in Jiangsu included Xuzhou, Lianyungang, Suzhou, Suqian, and Wuxi, with fewer cases reported in Yangzhou and Taizhou. Brucellosis cases were reported every year in Xuzhou, where the incidence is much higher than that in other regions for most years. In 2017, the incidence of brucellosis in Suzhou was the highest in Jiangsu, while from 2018 to 2020, Lianyungang's incidence surpassed that of Xuzhou, becoming the highest in the province. Figure 3 displays the temporal-spatial distribution map of the brucellosis incidence in Jiangsu Province from 2006 to 2021.

### Global spatial autocorrelation

The results of the global spatial autocorrelation analysis (conducted at the county level) of the annual incidence of brucellosis in Jiangsu Province from 2006 to 2021 showed that the values of Moran’s *I* were positive for 2008 and 2012–2021, (*|Z|* > 1.96, *p* < 0.05), indicating that there was no random distribution and a positive spatial correlation. Furthermore, the incidence of brucellosis exhibited an overall spatial aggregation distribution. The degree of aggregation was the highest in 2016 (Moran's *I* = 0.38) and lowest in 2019 (Moran's *I* = 0.12). There was no spatial autocorrelation in either 2006–2007 or 2009–2011 (*|Z|* < 1.96, *p* > 0.05), indicating no clustering trends during these years. Moran’s *I*, *Z* and *p*-value from 2006 to 2021 are listed in Table 1.

**Table 1:** Global autocorrelation of Moran’s *I* values of brucellosis in Jiangsu Province from 2006 to 2021.

Year	Moran's *I*	*Z* score	*p* value	Aggregation
2006	− 0.02	− 1.43	0.15	No
2007	− 0.03	− 0.37	0.71	No
2008	0.16	3.74	< 0.05	Yes
2009	0.00	0.26	0.79	No
2010	0.04	0.89	0.37	No
2011	0.04	1.33	0.18	No
2012	0.17	3.38	< 0.05	Yes
2013	0.16	2.91	< 0.05	Yes
2014	0.33	6.40	< 0.05	Yes
2015	0.30	5.40	< 0.05	Yes
2016	0.38	6.93	< 0.05	Yes
2017	0.31	5.79	< 0.05	Yes
2018	0.25	4.72	< 0.05	Yes
2019	0.12	2.53	< 0.05	Yes
2020	0.24	4.85	< 0.05	Yes
2021	0.34	6.42	< 0.05	Yes

### Temporal-spatial aggregation

**Figure 4:**
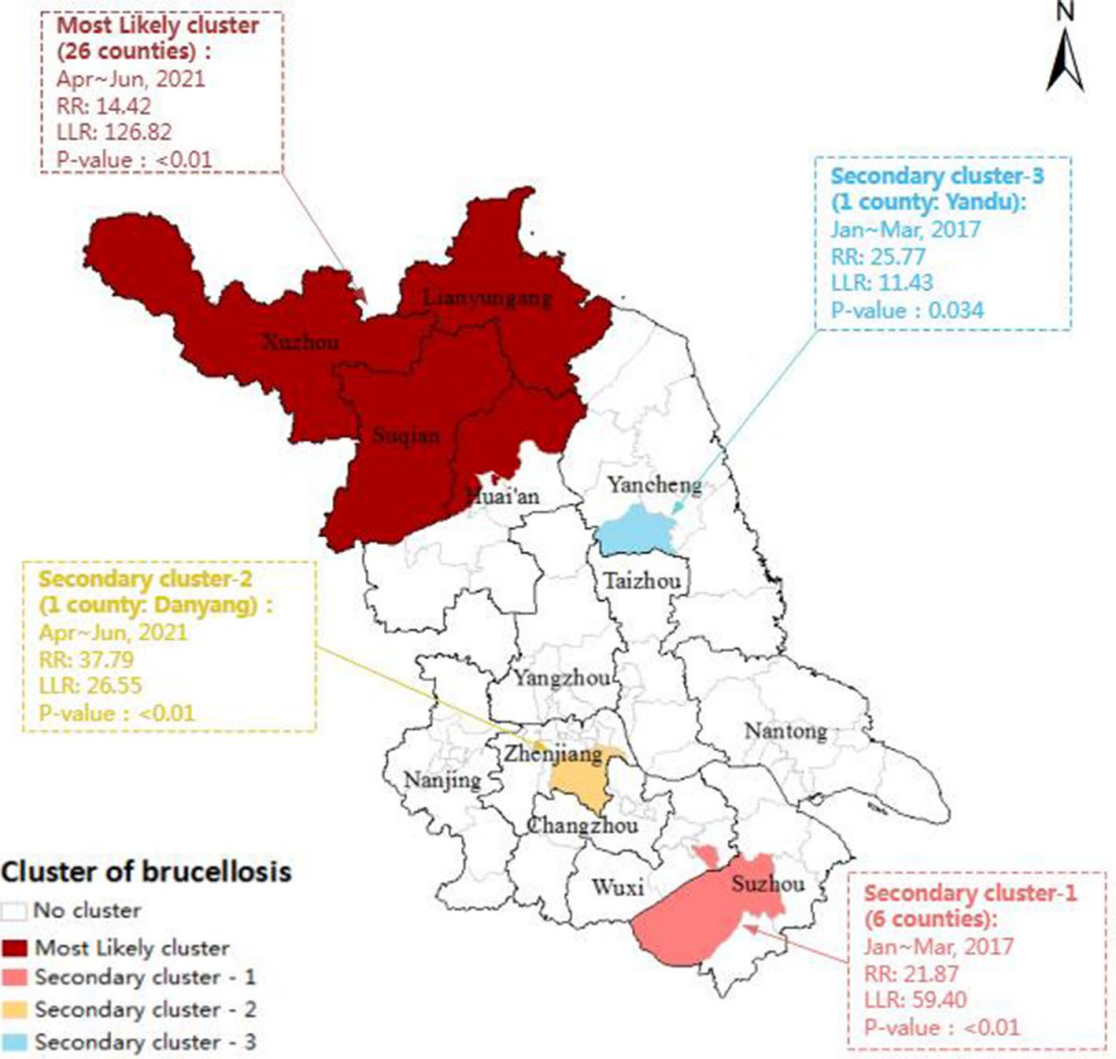
Temporal-spatial aggregation of brucellosis in Jiangsu Province from 2006 to 2021.

The results of the temporal-spatial clustering analysis showed that the clusters of the brucellosis epidemic in Jiangsu Province from 2006 to 2021 were mainly concentrated in both northern and southern Jiangsu. The time distribution of these clusters was consistent with the peak period of the brucellosis epidemic. These clusters included one most likely cluster and three secondary clusters. The most likely cluster was found in northern Jiangsu, including all 23 counties of Xuzhou City, Lianyungang City, Suqian City and three counties in Huai'an City, with a radius of 145.34 km, and the high-risk time frame was from April to June 2021 (LLR = 126.82, RR = 14.42, *P* < 0.01). Two of the three secondary clusters were distributed in southern Jiangsu: one was observed in Danyang County of Zhenjiang City, with a high-risk time frame spanning from April to June 2021; the other one covered Xinwu District in Wuxi City and some other counties in Suzhou, with a high-risk timeframe from January to March 2017. Another secondary cluster appeared in northern Jiangsu, covering the Yandu District of Yancheng City, with a high-risk timeframe from January to March 2017 (Fig. 4).

## Discussion

Brucellosis is a global issue of significant public health concern and deserves considerable attention for prevention and control in developing countries. Exploring the population, temporal and spatial aggregation and distribution of human brucellosis forms the basis for its control and elimination. This study provides theoretical support for the prevention and treatment of human brucellosis based on local conditions.

Our findings show a clear age-related difference in the incidence of brucellosis in the study population. Most of the reported cases in Jiangsu Province involve middle-aged and older adults (aged 40–69 years), and the majority were farmers. It is not difficult to see that the age distribution is based on the impotance of the family labour force. In rural Chinese families, most middle-aged and elderly people adults aged between 40 and 65 years are raising livestock or engaging in activities such as livestock trafficking, slaughtering, and fur, milk, and meat processing, especially in areas where livestock trading and slaughtering activities are more active^18–21^. This leads to a high incidence of brucellosis in this age group, suggesting that middle-aged and older occupational populations are at high risk of brucellosis infection. The presence cases among other age groups may be related to the consumption of unpasteurised milk^22^.

The brucellosis epidemic in Jiangsu Province is consistent with that in China. The incidence of brucellosis in Jiangsu Province increased rapidly from 2011 to 2021. The number of districts and counties reporting brucellosis cases increased from 13 in 2011 to 58 in 2017, eventually encompassing all cities in the province. The brucellosis epidemic in Jiangsu Province peaked in 2021, and the incidence of brucellosis in Xuzhou, Lianyungang and Suqian in 2021 was significantly higher than the average level in the previous three years (2018–2020). This was mainly due to the frequent mutton trading activities between these cities and endemic areas in northern Jiangsu in recent years. The importation of infected livestock increased the exposure risk of the occupational population, leading to an increasingly serious brucellosis epidemic in Jiangsu Province ^23^.

Seasonal decomposition showed that the incidence of brucellosis in Jiangsu Province increased annually from 2006 to 2021, and mainly concentrated from February to September, with fewer cases in other months. Meanwhile, it exhibited obvious seasonal characteristics, with a peak occurring from April to June. This is because the livestock trading and slaughtering market in Jiangsu Province is open from late October to the end of January of the following year. During this period, after exposure to infected animals, the occupational population usually begins to develop the disease in February after a certain incubation period (3 weeks–6 months, with an average of three months). In addition, the spring and summer seasons coincide with the delivery peak for sheep, cattle, and other livestock. Professional groups engaged in livestock delivery caring for sheep and cow calves are also prone to infection during this process ^24^.

The distribution of cases extended from northern and southern Jiangsu to central counties and districts such as Taizhou and Nantong. This is because Xuzhou in northern Jiangsu and Suzhou in southern Jiangsu have frequent human flow and livestock trading with neighbouring provinces (such as Suzhou in the north of Anhui and Hangzhou in Zhejiang), resulting in an increasingly serious epidemic of brucellosis in the north and south of Jiangsu, which gradually spread to the central area of Jiangsu^19,25^. These results indicate that the northern and southern regions of Jiangsu should further strengthen the prevention and control of brucellosis, including regulating livestock breeding and trading activities, and strengthening the quarantine of imported livestock. In addition, molecular epidemiological studies can be conducted to ascertain source of infection in brucellosis cases in the central region, enabling targeted prevention and control measures.

Global autocorrelation analysis revealed a positive annual spatial correlation between 2008–2012 and 2021 in Jiangsu Province with a clear spatial aggregation distribution at the county level. Spatial–temporal cluster analysis identified one primary cluster and three secondary clusters of human brucellosis incidence, which were concentrated in most parts of northern and southern Jiangsu, indicating that brucellosis propagates in a composite space–time domain rather than through pure spatial or temporal variation (see Fig. 4). Furthermore, the range of RR value of these four clusters was between 14.42 and 21.87, *p* < *0.05*, demonstrating that the risk of human brucellosis within these clusters is significantly higher than that in other areas of Jiangsu Province during the same period, which means that the clustering characteristics reflect the high incidence of brucellosis in Jiangsu.

The primary cluster was located in the areas of Xuzhou, Lianyungang, Suqian, and three other counties (Huaiyin, Lianshui, and Qinghe districts) in the northern Jiangsu. The clustering time was concentrated from April to June 2021. This clustering is attributed to frequent sheep trading activities between the above areas and the northern endemic areas of brucellosis, an increased risk of exposure to infected sheep through breeding, trafficking, and slaughtering activities, and the incidence of brucellosis in recent years^19,23,25^ indicating that prevention and control measures in these regions still need to be strengthened. One secondary cluster was identified in Suzhou from January to March 2017 and was caused by an outbreak of foodborne brucellosis in the main urban area of Suzhou in 2017^26^. The other two secondary clusters were identified in Danyang in Zhenjiang from April to June 2021 and the Yandu District of Yancheng from January to March 2017, where the professional population was infected by the imported brucellosis-infected livestocks due to insufficient personal protection during slaughtering activities^27^. Our findings indicated the link between highly epidemic areas and cluster characteristics^28^, particularly in non-endemic regions with high incidence rates. Therefore, dynamic changes in regions with a high incidence of brucellosis in Jiangsu Province may be closely related to the outbreak. Jiangsu has seen a rapid increase in brucellosis cases in recent years, with several outbreaks occurring at livestock slaughterhouses and trafficking hubs. We recommend that governments at all levels establish joint prevention and control mechanisms to implement strict brucellosis control measures in animals and humans to meet the requirements of disease control and prevention.

Our study has some limitations. First, cases that remained undiagnosed, were not reported, or did not meet the case definition criteria were not considered in this analysis, all of which would have led to an underestimation of the incidence of brucellosis in Jiangsu Province. Second, we used the adjacency criterion to carry out spatial autocorrelation analysis, and the size of the adjacent region scale was adjusted with the Wij (scale matrix), which could cause the change of correlation coefficient when Moran's *I* value is calculated, affecting the final aggregation result and causing a selection bias. Third, the method of spatial–temporal scan statistics used to detect clusters in different spaces and time periods relies only on circular space scans and cylindrical spatial–temporal scans and does not consider irregular spaces.

## Conclusion

This study helps identify populations, regions, and time frames at high risk for brucellosis, offering valuable insights for decision-making by relevant departments.

Regarding the distributional characteristics of the brucellosis epidemic in this study, we recommend enhancing detection and continuous monitoring efforts in northern and southern Jiangsu and applying effective strategies for brucellosis prevention and control in central Jiangsu, where the incidence of brucellosis is relatively low. Simultaneously, the capacity to respond to outbreaks should be improved to prevent brucellosis in areas with high incidence.

## Data Availability

The datasets used and/or analysed during the current study are available from the corresponding author on reasonable request.
